# Childhood Posterior Reversible Encephalopathy Syndrome: Clinicoradiological Characteristics, Managements, and Outcome

**DOI:** 10.3389/fped.2020.00585

**Published:** 2020-09-11

**Authors:** Tai-Heng Chen

**Affiliations:** ^1^Division of Pediatric Emergency, Department of Pediatrics, Kaohsiung Medical University Hospital, Kaohsiung Medical University, Kaohsiung, Taiwan; ^2^School of Post-Baccalaureate Medicine, College of Medicine, Kaohsiung Medical University, Kaohsiung, Taiwan; ^3^Section of Neurobiology, Department of Biological Sciences, University of Southern California, Los Angeles, CA, United States; ^4^Ph.D. Program in Translational Medicine, Graduate Institute of Clinical Medicine, Kaohsiung Medical University and Academia Sinica, Taipei, Taiwan

**Keywords:** posterior reversible encephalopathy syndrome, hypertension, seizures, neuroimgaging, childhood

## Abstract

Posterior reversible encephalopathy syndrome (PRES) is a novel clinicoradiological syndrome characterized by convulsions, headache, altered mentality, and impaired vision, which are usually accompanied by hypertension. As its nomination, PRES is usually diagnosed according to the presence of typical neuroimage showing vasogenic edema predominately involving the posterior brain area. With the widespread utilization of magnetic resonance imaging (MRI), PRES is becoming more perceptible in different medical fields. Compared to adult cases, childhood PRES seems to have a broader clinical and neuroradiological spectrum. PRES can be associated with various underlying comorbidities, medication use, and therapeutic modalities in children with diverse neurological manifestations. Moreover, pediatric patients with PRES have a more significant propensity for atypical MRI findings beyond the typically posterior cerebral areas. The knowledge of typical and atypical presentations in children is essential to avoid misdiagnosing or missing PRES, which is a potentially treatable entity. Early supportive care is the mainstay of treatment, with particular attention to the treatment of hypertension with rigorous attention to all body systems. Prompt identification and symptom-directed management are imperative to achieve a reversible prognosis in childhood PRES. Future studies specially designed for the child population are required to determine potential outcome predictors, and further, to develop novel strategies of neuroprotection in childhood PRES.

## Introduction

Posterior reversible encephalopathy syndrome (PRES), initially reported from 15 adult patients by Hinchey et al. (1), is a well-recognized entity characterized by a combination of clinical and radiological features. This conditionally induced neurological disorder has been indicated by different terms, including reversible posterior leukoencephalopathy syndrome, reversible occipital-parietal encephalopathy, and reversible posterior cerebral edema syndrome. Although PRES is the generally acknowledged term for its clinical and radiological reversibility, it has been continuously criticized due to the potential risks of permanent neurological sequelae and as high as 16% of the mortality rate (2, 3). Clinically, PRES is featured by the diversity of seizure patterns, headache, clouding of consciousness, impaired vision, and sometimes, and focal neurological defects. Besides, the diagnosis of PRES is always accompanied by peculiar radiological findings of edematous change affecting the rear cerebral area (4).

With the global application of magnetic resonance imaging (MRI), PRES is becoming more recognizable to physicians, particularly radiologists and neurologists (5). Besides, a broad spectrum of clinical and radiological patterns has emerged with increasing reports. However, since there are relatively few literature reports on pediatric PRES cases and their appearance is relatively late compared with adults, pediatricians seem to have little experience with the disease.

Although PRES is increasingly diagnosed in adults and children, it is often overlooked by the first-line physicians in most cases, and radiologists may be the first to notify the diagnosis. It has been known that PRES-inducing disorders can vary in different age groups. Indeed, there are several critical differences between the children's brain and an adult one, such as vulnerability to noxious substances, regulatory ability in cerebral hemodynamics, and vasoregulation. Also, regeneration potential, radiological picture, and the clinical course of PRES might differ among different age groups (6). In this review, we will discuss the pathophysiology, clinicoradiological characteristics, diagnostic approaches, treatment, prognostic factors, areas of uncertainty, and future perspectives of management in childhood PRES. As the spectrum of features characterizing this neurological disorder is broader than usually expected, the objective of this review is also to compare the distinctness in clinicoradiological features between adult and childhood PRES.

## Epidemiology

After its initial description in 1996, the identification of PRES induced by various etiologies has shown exponential growth over the past few decades. These reports are in the form of single case reports, case series, broad-scale retrospective researches, and meta-analysis systemic reviews from large institutions. The epidemiological data come from retrospective studies of adult groups, and some from pediatric groups have reported. A broad age range, from 4 to 90 years, has been reported to susceptible to PRES, but the majority of patients are young to middle-aged adults, with a mean of 39 to 47 years documented in several case series (4, 7–9). There is a marked female predominance in the patient population. This finding implies that some underlying comorbidities related to PRES are gender-specific. Although the exact incidence of PRES in children is not known, it is estimated to be rare. Most data on childhood PRES comes from different single-center retrospective studies and focuses on a specific subset of a pediatric group. The roughly estimated incidence of PRES in the general pediatric population is 0.04% (8), 0.7% of children with cancer, and 0.4% of children with admission to the pediatric intensive care unit (PICU) (10). In a review regarding the pediatric recipients of autologous hematopoietic cell transplantation, PRES was documented in 5.2% of children with complications (11). An updated nationwide survey reported the mean age at presentation more common in the adolescent age group (mean: 12.5 years) (8). In earlier researches, the mean age of pediatric oncology patients complicated by PRES ranged between 7 and 9 years, with females predisposed to have a higher risk (12).

Quite a few children with PRES have underlying disorders, including renal diseases, systemic lupus erythematosus (SLE), sickle cell disease, and bone marrow or solid organ transplantation (1, 9). In particular, renal insufficiency of various etiologies, followed by hematologic diseases and related therapies, including cytostatic agents of corticosteroid (for malignancy or immune suppression), are the most common conditions triggering PRES in childhood (8, 13, 14). Several chemotherapeutic agents and total body irradiation have been reported to trigger PRES in children. Table 1 summarizes the underlying disorders and toxic agents known to precipitate PRES in the pediatric population.

**Table 1:** Risk factors for posterior reversible encephalopathy syndrome in children.

**Hypertension**
Primary (Idiopathic) Secondary: (More in Children) Renovascular dysplasia, Pheochromocytoma, Ganglioneuroma, Primary aldosteronism, Acute/chronic kidney disease, Hyperthyroidism, Drugs (e.g., Amphetamine, cocaine)
**Renal disorder**
Glomerular disease, Tubulointerstitial disease, Henoch–Schönlein purpura
**Collagen vascular disorders**
Systemic lupus erythematosus, Polyarteritis nodosa, Behçet's syndrome
**Sickle cell anemia**
**Following solid organ or bone marrow transplantation**
**Acute intermittent porphyria**
**Thrombotic-thrombocytopenic purpura**
**Acquired immunodeficiency syndrome**
**Use of immunosuppressive agents**
Cyclosporine A, Tacrolimus, Azatioprine, Rapamicine, Sirolimus, High-dose corticosteroid therapy (e.g., dexamethasone and methylprednisolone)
**Cancer chemotherapy agents (in combination)**
**Using cytotoxic agents**
Alkylating agents: Cisplatin, Oxaliplatin, Carboplatin Antimetabolites: Gemcitabine, Cytarabine, Methotrexate, Fludarabine Mitotic inhibitors: Vincristine, Irinotecan hydrochloride Others L-asparaginase
**Monoclonal antibodies**
Rituximab, Infliximab, Alemtuzumab
**Immunomodulatory cytokines**
Interferon-α, Interleukin-2
**Antibiotics**
Linezolid, Ciprofloxacin
**Growth factors**
Granulocyte-stimulating factor, Erythropoietin
**Intravenous immunoglobulins**
**Blood transfusion**
**Miscellaneous**
Intravenous contrast agents, Carbamazepine, Epinephrine

## Potential Pathomechanisms of PRES

Till now, the pathomechanism underlying PRES is yet to be thoroughly elucidated. Two competing theories have been proposed, both of which entail disruption of the blood-brain barrier and fluid leakage into the interstitial tissues, leading to the edematous change of cerebral parenchyma (9, 15, 16). However, more evidence indicates that vasogenic edema rather than cytotoxic edema plays a more critical role in the pathogenesis of PRES (17, 18). The first putative pathophysiological principle is impaired cerebrovascular auto-regulation in combination with endothelial dysfunction, which leads to temporarily vasogenic edema of the cerebral parenchyma. The vasogenic mechanism presumes that hypertension may surpass the limit of cerebrovascular auto-regulation, partially through endothelial overstress, then failing compensatory vasoconstriction to restrain hyperperfusion of cerebral blood flow. Especially the elevation in blood pressure is so dramatic that the under-reactive autoregulatory response of the cerebrovascular system may lead to hyperperfusion and subsequent leakage of plasma and macromolecules from vessels. The preferential involvement of the parietal-occipital regions is considered to be due to fewer sympathetic innervations of vessels that originate from the vertebrobasilar circulation when compared with the carotid system (17, 19).

However, 15–20% of patients with PRES have normal or only slightly high blood pressure (20). Therefore, another hypothetic potentiating PRES pathomechanism accordingly refers to the cytotoxic effect by which several anti-neoplastic and immunosuppressive agents cause direct destruction to the cerebrovascular endothelium (21). The endothelial dysfunction can result in vasoconstriction of microvasculature, which might be further aggravated by hypertension and dysregulated autoregulatory response (22, 23). Indeed, as some patients with sepsis and hypotension can also develop PRES, it has been postulated that marked fluctuations in blood pressure, instead of absolute blood pressure elevation, might play a more significant role in precipitating the syndrome (24). Another intriguing hypothesis of the pathomechanism for PRES-associated cerebral edematous change addresses the role of vascular endothelial growth factor (VEGF), which is involved in regulating the permeability of the endothelial barrier. The dysregulated level of VEGF has been associated with several conditions characterized by leakage of vessel fluid (23). In patients with autoimmune diseases, antigen-antibody interaction, and its associated aberrant inflammations may also contribute to the endothelial disruptions (25).

Compared with adults, under systemic hypertension, children may be more likely to suffer from cerebrovascular dysregulation than adults, because the range of auto-regulation of cerebral blood flow is relatively narrow (6, 26, 27). Although the thresholds vary among individuals, the lower limit of cerebral blood flow auto-regulation is approximate 50–60 mm Hg in adults, but the average level is as low as 40 mm Hg in children (28). Furthermore, a retrospective study showed that the mean blood pressure was 140/85 mm Hg in pediatric patients at the onset of PRES (27). Thus, children seem to be more susceptible to PRES, and its prevalence could be under-estimated (13). In a large-scale review of pediatric patients who had undergone oncological therapies, hypertension-related PRES was identified in 88% of cases (21). Among children admitted to the PICU, 80% of PRES cases were documented as hypertensive status (blood pressure exceeded 99th percentile of the normal range) for at least 6 h before the onset of PRES-related symptoms/signs (10). It has been suggested that aggressively lowering blood pressure can prevent pathological changes from benign vasogenic edema to complicated cytotoxic edema, cerebrovascular infarctions, and even irreversible neurological deficit (29).

## Clinical and Radiological Characteristics of PRES

### Typical and Novel Manifestations of Childhood PRES

As shown in Table 2, the clinical manifestations in PRES are often non-specific and can be encountered in many other neurological diseases (1, 13). The onset of symptoms is usually prompt and peaks within 12 to 48 h (18). The majority of PRES-related literature comes from retrospective-design observational studies, and the frequency of these symptoms depends on the sample size and predisposing factors evaluated. In general, seizures have been documented as the most common manifestation in childhood PRES. The patterns of seizures are usually of generalized tonic-clonic (GTC) type (about 60–75% of patients) and may be preceded by visual impairment, consistent with its origin at the affected occipital lobes (25, 26, 30). The GTC seizures are usually multiple rather than a single episode, and sometimes a status epilepticus may develop (31). It is worth noting that as an initial PRES-related symptom, seizures in childhood PRES seem to be more common and onset earlier than adult patients (26). An electroencephalogram (EEG) usually reveals non-specific encephalopathic changes reported in 64% of children with PRES (6, 27). However, PRES may be suspected as the underlying cause of status epilepticus when bilateral occipital sharp waves are present on EEG (15, 18).

**Table 2:** Clinical symptoms and signs in children with posterior reversible encephalopathy syndrome.

Hypertension
Seizures
Altered mental state, ranging from lethargy to coma
Headaches
Visual disturbances, ranging from hemianopsia to anopsia
Nausea/vomiting
Focal neurological deficit, including hemiparesis
Status epilepticus
^*^Rare: back/nuchal pain, lower legs weakness, incontinence

* *When spinal cord was involved, namely posterior reversible encephalopathy syndrome with spinal cord involvement (PRES-SCI)*.

Besides seizures, altered consciousness was commonly found in more than half of pediatric patients (26). According to previous reports, 28–94% of adult PRES patients have varying degrees of encephalopathy, ranging from mild confusion, cognitive impairment, somnolence, stupor, and coma (15). In some adult PRES cases, the incidence of acute encephalopathy is even higher than that of seizures, being 92 and 87%, respectively (7). Hence, it has been suggested that an EEG should be promptly obtained in patients suspected to have PRES with unexplained altered consciousness (18). However, altered mental status is reported in only 52% of pediatric PRES cases. Furthermore, it seems that visual impairment occurring more frequently in adults than in children (6).

As non-specific neurologic features are usually of little diagnostic value for PRES, hypertension, which presents in 70–80% of these patients, always warrants a diagnostic hallmark of PRES (1, 6). In the pediatric population, it has been suggested that 30% above the 95 percentile of blood pressure for age should potentiate a clinical alert for the PRES onset (32). Correspondingly, a systematic review of pediatric groups with PRES indicates that 85% of children are hypertensive, suggesting an essential role of hypertension playing in the pathomechanism of childhood PRES (26). Childhood hypertension is usually associated with a systemic disorder, including renal parenchymal disease, pheochromocytoma, adrenal disorders, primary renovascular dysplasia, or endocrine abnormalities (Table 1). In contrast to adults, childhood hypertension is frequently overlooked, particularly in the scenario of a neurological emergency such as extreme irritability, altered consciousness, or acute seizures (13, 28). Otherwise, elevated systemic blood pressure associated with agitation or the seizure-induced hyper-sympathetic tone might also result in the evaluation of neurological disorders with systemic problems like hypertension being overlooked in a child (26, 33).

Moreover, systemic hypertension is always regarded as a temporary compensation for increased intracranial pressure, instead of the culprit causing acute encephalopathy. Hence, it is common that children have been identified hypertensive only late in the clinical course of PRES (10, 27, 34). Our previous experience addressed the importance of meticulous surveillance of blood pressure in children presenting with critically neurological manifestations. We suggested that pediatricians should put PRES into a list of differential diagnoses, particularly when hypertension is found in the context of sudden altered mental status or seizures (26, 35).

### Typical and Atypical Neuroradiology Features of Childhood PRES

As being a clinicoradiological entity, brain imaging is informative to exclude alternative diagnoses from PRES; therefore, being thoroughly conversant with the neuroimaging criteria is essential to the precise diagnosis (9, 15). The combination of suggestive clinical and radiological characteristics helps to confirm a definite diagnosis of PRES. PRES is typically associated with edematous changes in a bilaterally symmetric pattern, which typically located in the subcortical areas of the parieto-occipital lobes (36). Neuroimaging usually depicts a relatively symmetrical and extensive range of abnormalities within 24 h of clinical onset. The identification rate of abnormalities on initial computed tomography (CT) and MRI is 46 and 98%, respectively, in the childhood PRES (26). Although brain CT might sporadically identify lesions with hypodensities, it is not reliable to confirm PRES (13). Therefore, MRI, including various weighted imaging, could represent a useful tool to diagnose PRES because of its high sensitivity and specificity (4).

**Figure 1:**
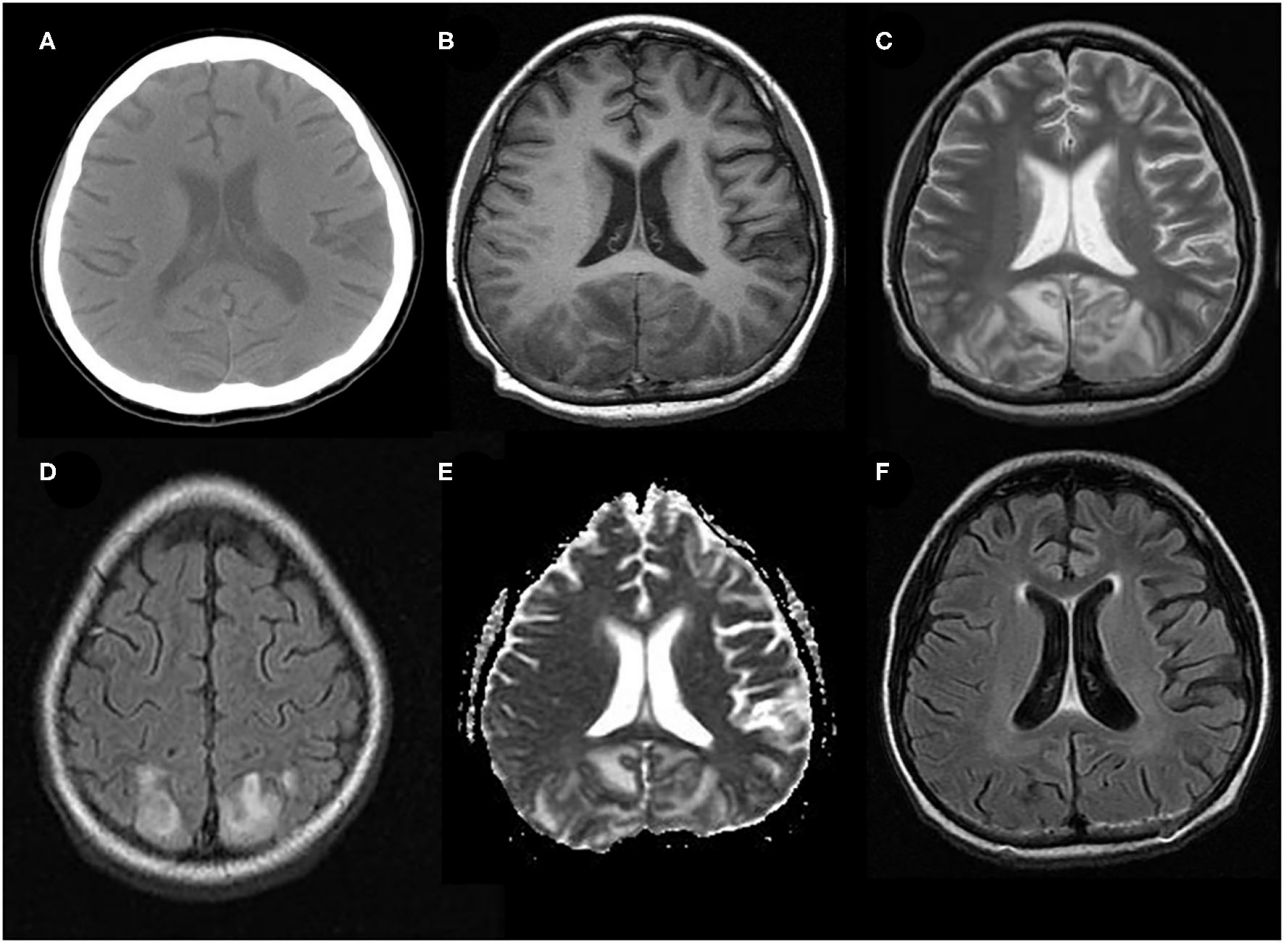
Typical neuroimaging of PRES in a 9-year-old male with underlying nephritic syndrome. **(A)** Initially, unenhanced brain CT showed mildly cortico-subcortical hypodensities, particularly in bilateral parieto-occipital regions. **(B)** At the acute stage, these cerebral lesions showed hypointensities on T1-weighted MRI in bilateral parieto-occipital regions **(C)** T2-weighted MRI, and **(D)** T2-FLAIR MRI showed symmetrical hyperintensities in bilateral parieto-occipital regions. **(E)** ADC map shows a hyperintense signal in the aforementioned corresponding areas. **(F)** FLAIR images 2 months later, showing a complete resolution of the abnormal signal.

**Figure 2:**
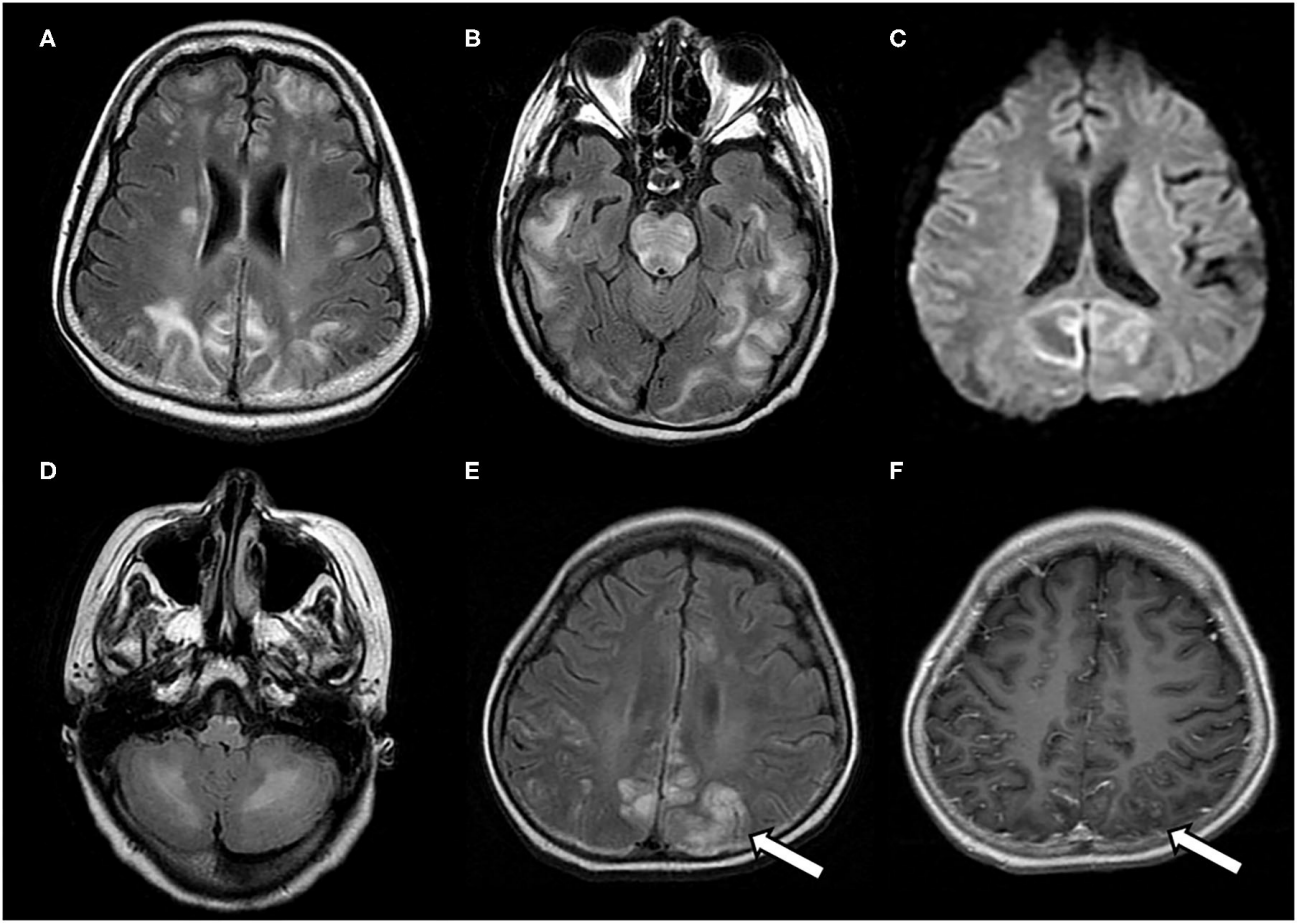
Atypical neuroimaging findings of PRES. **(A,B)** In a 17-year-old female with underlying systemic lupus erythematosus, lesions diffusely involving cerebral hemispheres, basal ganglia, and brain stem, with a prominent asymmetry on FLAIR images. **(C)** In a 12-year-old female with nephritic syndrome, MRI showed paradoxically hyperintense lesions on DWI (restricted diffusion) in bilateral occipital lobes. **(D)** In a 14-year-old male with membranoproliferative glomerulonephritis who showed prominent lesions of bilateral cerebellar hemispheres in FLAIR images **(E,F)** In an 8-year-old male with nephrotic syndrome, MRI showed occipital lesions hyperintensities on FLAIR, which also demonstrate post-contrast enhancement on T1-weighted image (white arrows).

The typical MRI findings of PRES usually show abnormal signals with hyperintensities on T2-weighted MRI and fluid-attenuated inversion recovery (FLAIR) imaging, accompanied by the increased value of apparent diffusion coefficient (ADC) (Figure 1) (1, 4, 18, 37). In the same areas of lesion, T1-weighted images usually illustrate hypointense foci, with normal diffusion-weighted imaging (DWI) (17). Lesions usually distribute in both cortical and subcortical areas of the posterior brain region (parieto-occipital lobes). These imaging features represent vasogenic edema, which mainly contributes to the classical theory of PRES pathomechanisms. Otherwise, atypical MRI features of PRES, as shown in Figure 2, are usually identified in the following cerebral regions with several radiological characteristics, include (1) location in the frontal lobes, basal ganglia, corpus callosum, cerebellum, and brainstem; (2) gadolinium enhancement; (3) presence of hemorrhagic changes; and (4) restricted diffusion showing in ADC or DWI (7, 17, 36–38). These atypical MRI features can demonstrate cytotoxic edema caused by acute ischemia leading to reduced ADC but increased DWI signals, namely restricted diffusion. Interestingly, partial or asymmetric expression in either the parietal or the occipital lobes seems more common than the classical parietal-occipital involvements in adult PRES patients (4).

Of note, PRES may be complicated by a cerebral hemorrhage, and its frequency may be underestimated (30, 38). A new technique called susceptibility-weighted imaging (SWI) is more sensitive than conventional T2 gradient recalled echo (T2^*^GRE) imaging in detecting cerebral hemorrhages in PRES (39). SWI may have additional benefits as it can help to identify either frank intraparenchymal hemorrhage or even early microhemorrhages associated with PRES (38). Although the clinical relevance of microhemorrhages in PRES has not yet been determined, larger intraparenchymal hemorrhage may potentially develop into sequela and affect the prognosis of PRES (40).

Atypical MRI lesions are reported between 61 and 82% of children (14, 26, 34), compared with 10–58% in adult patients (1, 7, 38, 41) In childhood PRES, the superior frontal sulcus involvement is common and is even more frequently seen in children than in adults (26, 34). Besides, MRI features of restricted diffusion are observed in 15–42% of childhood PRES, compared to 15–30% of adulthood PRES (42). The pathomechanisms underlying this difference are not yet fully clarified and would warrant further investigation. Therefore, it has been underscored that the high prevalence of atypical neuroimaging features in childhood PRES may make the diagnosis more complex in children than in adults.

## Differential Diagnosis of Childhood PRES

The differential diagnosis of PRES includes many possibilities (9, 18). Differentiating PRES from other acute neurological disorders in children usually requires a thorough review of medical history, neuroimaging, and additional laboratory investigations. Table 3 lists the differential diagnosis of PRES encountered explicitly in the pediatric population. Even though PRES is a relatively rare entity, the broad clinical presentations and multiplicity of neuroimaging patterns produce diagnostic challenges. Because the clinical features of PRES are usually unspecific, radiological characteristics of brain MRI are indispensable to preclude other possible diagnoses. Nevertheless, the diagnosis of PRES cannot always possibly rely on single neuroimaging findings, and the clinical background judged by first-line clinicians is essential to make an accurate diagnosis.

**Table 3:** Differential diagnoses of posterior reversible encephalopathy syndrome in children.

Infectious encephalitis
Autoimmune or paraneoplastic encephalitis
Malignancy or Neoplasms (lymphoma, gliomatosis cerebri, metastatic disease)
Cerebral venous thrombosis
Top of basilar syndrome
Severe subcortical leucoaraiosis
Posttransplant lymphoproliferative disease
ictal or post-ictal state (with or without status epilepticus),
Progressive multifocal leukoencephalopathy (PML)
Acute disseminated encephalomyelitis (ADEM)
Cerebral autosomal dominant arteriopathy with subcortical infarcts and leukoencephalopathy (CADASIL)
Ischemic stroke (watershed or posterior cerebral artery territory)
Mitochondrial myopathy encephalopathy lactacidosis and stroke-like episodes syndrome (MELAS)
Vasculitis of central nervous system
Osmotic demyelination syndrome
Toxic leukoencephalopathy

## Monitoring and Management of PRES in Pediatric Cases

To date, no randomized controlled trials assessing therapeutic interventions have been conducted in patients with PRES. Therefore, no specific treatment is currently available for PRES, and supportive care is the mainstay (15, 18). Each subject should be treated individually based on their clinical conditions, related risk factors, and tolerable pharmacotherapy. Early etiologic identification allows timely correction of the cause of PRES. The symptom-directed management is imperative and usually comprises antihypertensive agents, anticonvulsive agents, removal of hazardous agents, and correction of the underlying comorbidities inducing hypertension (9). Sophisticated correction of the underlying causes can help to decrease the risk of irreversible complications of PRES, such as cerebral ischemia or hemorrhage, and, therefore, to avoid permanent disability or death.

Prompt control of any type of seizures is imperative, and continuous surveillance of EEG is encouraged to evaluate the subtle seizures. The requirement for upper airway protection should be evaluated continuously in children with markedly compromised consciousness or prolonged seizures. Patients with repeated seizure activity can be administered intravenous benzodiazepine, with repeated doses up to three times if needed. Persistent seizure activity, which is refractory to the highest dosage of intravenous benzodiazepines, should be treated with second-line antiepileptic drugs, such as phenobarbital, phenytoin, or equivalent dose of fosphenytoin (9, 18). Nevertheless, even though the administration of antiepileptic agents is essential for the acute management of PRES-related seizures, this does not necessitate long-term antiepileptic use (7, 15). According to a single-center study analyzing the risk of subsequent seizures following resolution of PRES, the median duration of treatment with antiepileptic drugs (mainly levetiracetam and phenytoin) was 3 months (IQR 2–7 months) (43).

Severe hypertension should be closely monitored and treated aggressively (14, 15, 26). Lowering blood pressure seems to be more well-tolerated in children except in the setting of chronic hypertension or cerebral vasculopathy. It should be kept in mind that the treatment goal is not to normalize the blood pressure but rather to gradually lower the mean arterial pressure by 20–25% within the first 2 h (9, 18). Subsequently, one-third of the total planned blood pressure reduction is targeted during the first 6 h, another third during the next 24 to 36 h, and the final third during the next 24 to 96 h or even longer (15, 44). An abrupt reduction in blood pressure should be avoided because cerebral hypoperfusion might occur. In the treatment of hypertensive emergency, sodium nitroprusside is one of the most commonly used drugs with acceptable safety in children. Also, labetalol, nicardipine diazoxide, hydralazine, esmolol, enalaprilat, and nifedipine are known to treat hypertensive emergencies safely in children (26, 27). Besides pharmacological control of hypertension, children with PRES should be treated clinically with careful attention to the management of minimizing the effects of increased intracranial pressure. Standard practice measures might also include rising the head of the bed to 30–45 degrees, maintaining normoxemia, and avoiding hypercarbia (10).

It is important that for children with renal insufficiency or failure, the administration of medications for the management of PRES should be based on renal function. Care should be taken to correct abnormal serum electrolytes and pay close attention to sodium, calcium, and magnesium (9, 18, 26, 27). Although this has not yet been extensively studied and the risk is unclear, it is usually possible to continue chemotherapy after hypertension is resolved. Approximate 35–40% of patients with PRES may require mechanical ventilator support for 3 to 7 days due to severely compromised consciousness or refractory seizures (7, 45).

Nevertheless, patients with PRES always require management in the setting of intensive care units given that hypertensive crisis, status epilepticus, coma, or respiratory failure may complicate PRES, which require aggressive interventions (7, 15, 18). In adult medicine, it has been suggested that the management of PRES should be better delivered in a neurocritical care setting with meticulous neurological monitoring and interventions (9, 16, 46). However, besides neurological specialty, it usually requires frequent consultations with other specialties, including hematologist-oncologist, nephrologists, rheumatologists, or obstetricians, according to the diverse underlying comorbidities (31). On the other hand, it has been suggested that the advantages of multidisciplinary care in the PICU settings, which may have contributed to a favorable outcome in childhood PRES. In our previously reported case series (26), the median time of clinical resolution in children with PRES is 4.8 days (range: 1.5–14 days), that is compatible with the adult patient group (5.3 days) (7).

Recently, it has been postulated that the pathomechanism of PRES may be related to the activation of the arginine vasopressin (AVP) axis by increasing AVP secretion or increasing AVP receptor density (47). Accordingly, suppression of AVP hypersecretion and/or of its pharmacologic effects by antagonizing AVP receptors might be a promising therapeutic approach for PRES. The most attractive approach would be to combine both reductions of AVP secretion and pharmacological blocking of its effectors, for example, cerebral AVP receptors (V1aR) and peripheral (renal) receptors (V2R) (15, 47).

## Complications and Variants of Childhood PRES

The complication of cerebral infarction, which is present at the acute phase of PRES in about 10-23% of patients with available DWI, is among the early signs of irreversible damage associated with unfavorable outcomes (4). Accordingly, a prompt differentiation between PRES and ischemic stroke, which may resemble each other in neuroimaging findings, is crucial in managing PRES (15, 17, 38). Prompt control of acute hypertension is mandatory in the context of PRES; however, if ischemic infarction is complicated, a higher systemic BP would be kept in order to maintain adequate cerebral blood flow, and sometimes, a further institution of anticoagulants might also be ordered. Unfortunately, such infarction-directed management could worsen the clinical course of PRES, and a catastrophic consequence of intracranial hemorrhage has been reported in PRES patients with such misled management (48). Indeed, cerebral hemorrhages are rare complications of PRES, with about 5–17% of adult patients who may present with parenchymal or subarachnoid hemorrhages (17, 36). Patients receiving autologous bone marrow transplantation or anticoagulant treatment may have a higher risk of cerebral hemorrhage; however, blood pressure does not correlate to the bleeding risk (9, 30). As aforementioned, utilization of the novel SWI technique can help to detect PRES-related hemorrhage, particularly in the early stage of microhemorrhages (39).

Diagnosis of PRES could be challenging in children with preexisting SLE, because of the shared clinical pictures of acute encephalopathy between PRES and neuropsychiatric lupus, which occurs in 25% of SLE patients (49). However, the treatment of PRES is mainly supportive, different from immunosuppressive treatment or other specific treatment of neuropsychiatric complications of SLE (50, 51). Treatments of lupus-associated CNS involvement consisting of pulse therapy of corticosteroid, and immunosuppressants of cyclophosphamide, and cyclosporine, which are known offensive agents for PRES and might further worsen the outcome of PRES (1, 49).

**Figure 3:**
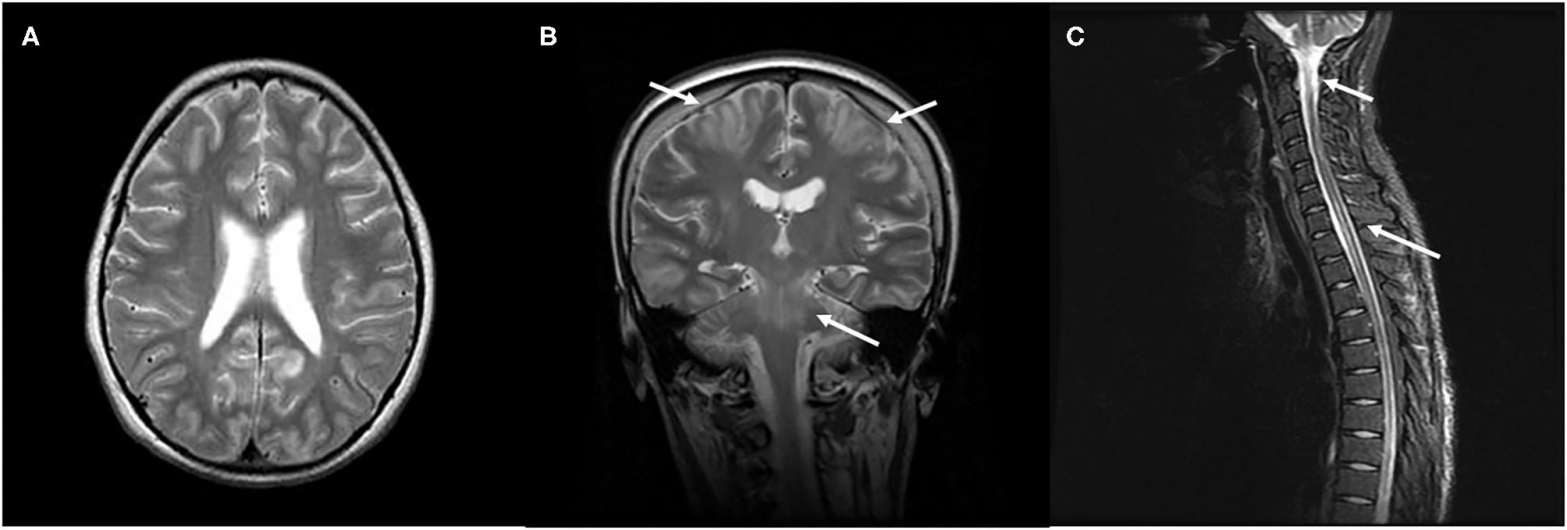
Characteristic neuroimaging findings of a 15-year-old male with posterior reversible encephalopathy syndrome with spinal cord involvement (PRES-SCI). **(A)** Axial view of brain MRI showing T2-weighted hyperintensity in the bilateral parieto-occipital and frontal lobes. **(B)** Coronal view of MRI showing T2 hyperintensity in the brain stem, as well as in bilateral frontoparietal lobes (white arrows). **(C)** Sagittal cervicothoracic spine demonstrating longitudinally T2-weighted hyperintensity originating at cervicomedullary junction (white arrows).

Recently, a variant of PRES with spinal cord involvement (PRES-SCI) has been reported in some adult patients (52). Subsequently, few pediatric cases have been increasingly reported (53–55). Besides typical involvements of parieto-occipital regions in the brain, these patients also show a diffusely longitudinal central-cord edema without evidence of infection, demyelination, or ischemia (Figure 3). The prevalence of PRES-SCI is unclear; to our knowledge, 19 patients, including eight children, have been systemically analyzed (52, 56). There are several clinical features distinct pediatric cases from adult ones. Contrary to adults, children with PRES-SCI usually have less severe hypertension, but a higher rate of atypical neuroimaging presentations and abnormal cerebrospinal fluid (CSF) findings, and a higher chance of uneventful recovery (56). Awareness of this rare features of the spinal variant of PRES in the pediatric group is imperative to prevent an unnecessarily invasive intervention and to obtain a favorable outcome.

## Prognosis of Children with PRES

Generally, PRES is regarded to be both clinically and radiologically reversible and usually has a favorable prognosis while it is treated timely, with uneventful recovery within days or weeks (15, 16, 18, 26, 27). Clinical manifestations usually clear progressively within four days, but they may persist for over a few weeks. A previous study reported that repeat MRI showed complete or near-complete resolution of cerebral edematous changes in 85% of children with PRES at a median follow-up of 29 days (27). A transient relapse is possible 1–2 weeks later, in 5–10% of patients, especially in those with poor control of hypertension (57). In general, the recurrence rate of PRES is between 3.8 and 12.5%, while the recurrence rate of the pediatric population seems to be higher (7, 58). Previous studies have also indicated that repetitive hypertension in the setting of chronic renal disease is the most common etiological factor of the first episode and recurrent PRES in the pediatric population (14, 58, 59). Despite a high frequency of provoked seizures during the acute phase of PRES, the long-term risk of unprovoked seizures is infrequent, and epilepsy is rare (43).

However, delayed or misdiagnosis, followed by improper management, may lead to irreversible damage to affected neuronal tissues or even death (9, 15, 18, 60). Failure to timely eliminate the causative factor in PRES could also represent a significant predictor of adverse outcomes, which might further explain the seemingly worse prognosis of these patients (61). The level of consciousness and complications of subarachnoid hemorrhage, as well as laboratory findings, including elevated C-reactive protein and impaired coagulation profiles, are prognostic factors associated with outcome in adult PRES (62). On the other hand, an updated study indicated that the underlying end-stage renal disease, presence of status epilepticus, PICU history, atypical MRI lesions, and increased inflammatory markers might be potential prognostic factors of childhood PRES (14). Even though neuroradiological abnormalities are thought to be reversible, some patients may be left with permanent cerebral lesions and even brain atrophy. Hippocampal sclerosis has been reported in patients who developed temporal lobe epilepsy months to years after PRES episodes (63).

Abnormal MRI findings in late stages of children with PRES were associated with neurological sequelae (64). Several adult studies indicate that distribution and MRI signal intensity of affected brain lesions do not correlate to the underlying comorbidities, clinical severity, or the degree of hypertension (9, 15). Specifically, increased DWI signals have been suggested to be associated with a more unfortunate outcome in adult PRES patients (41, 65). However, the MRI diffusion sequence could be misleading, as both increased and decreased values may be incidental findings with no prognostic value (17, 25, 38). On the other hand, elevated serum lactate dehydrogenase levels (LDH) and C-reactive protein (CRP) have been proposed as potential biomarkers to predict the outcome in some patients with PRES; however, the most findings were based on a small group of patients (16, 62, 66, 67). The role of clinical, biochemical, and radiological parameters in predicting the prognosis of patients with PRES warrants further verification.

## Conclusions

PRES may complicate a diversity of underlying comorbidities among the child population. This review summarized recent progress in the diagnosis, understanding of pathomechanism, and management of childhood PRES. We suggest that, in children presenting with new-onset seizures or altered consciousness accompanying systemic hypertension, PRES should be considered within a comprehensive differential diagnosis of acute encephalopathy. Careful assessments are imperative, including continued monitoring of blood pressure and appropriate neuroimaging investigations. Early recognition and multidisciplinary management are essential to achieve a reversible outcome. A standardized algorithm that integrates the clinical, etiological, serum/CSF biomarkers, and radiological characteristics of diverse underlying comorbidities will help future research. It would be valuable to explore various pathomechanisms of PRES at the bench side to identify reliable laboratory and imaging markers and potential therapeutics to improve functional outcomes. Future studies specially designed for the pediatric population are needed to identify risk factors associated with adverse outcomes and to develop novel strategies of neuroprotection in childhood PRES.

## Author Contributions

T-HC contributed to conception and design, acquisition of data, revising the manuscript critically for relevant intellectual content, final approval of the version to be published, read and approved the final manuscript.

## Conflict of Interest

The author declares that the research was conducted in the absence of any commercial or financial relationships that could be construed as a potential conflict of interest.
